# Functionalized monodisperse microbubble production: microfluidic method for fast, controlled, and automated removal of excess coating material

**DOI:** 10.1038/s41378-024-00760-y

**Published:** 2024-08-30

**Authors:** M. R. P. van den Broek, M. Versluis, A. van den Berg, T. Segers

**Affiliations:** 1 BIOS/Lab on a Chip Group, Max Planck Center Twente for Complex Fluid Dynamics, University of Twente, Enschede, The Netherlands; 2 Physics of Fluids Group, University of Twente, Enschede, The Netherlands

**Keywords:** Engineering, Physics

## Abstract

Functionalized monodisperse microbubbles have the potential to boost the sensitivity and efficacy of molecular ultrasound imaging and targeted drug delivery using bubbles and ultrasound. Monodisperse bubbles can be produced in a microfluidic flow focusing device. However, their functionalization and sequential use require removal of the excess lipids from the bubble suspension to minimize the use of expensive ligands and to avoid competitive binding and blocking of the receptor molecules. To date, excess lipid removal is performed by centrifugation, which is labor intensive and challenging to automate. More importantly, as we show, the increased hydrostatic pressure during centrifugation can reduce bubble monodispersity. Here, we introduce a novel automated microfluidic ’washing’ method. First, bubbles are injected in a microfluidic chamber 1 mm in height where they are left to float against the top wall. Second, lipid-free medium is pumped through the chamber to remove excess lipids while the bubbles remain located at the top wall. Third, the washed bubbles are resuspended and removed from the device into a collection vial. We demonstrate that the present method can (i) reduce the excess lipid concentration by 4 orders of magnitude, (ii) be fully automated, and (iii) be performed in minutes while the size distribution, functionality, and acoustic response of the bubbles remain unaffected. Thus, the presented method is a gateway to the fully automated production of functionalized monodisperse microbubbles.

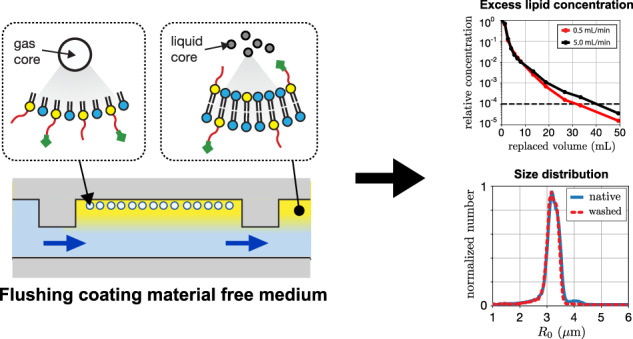

## Introduction

Ultrasound is one of the most widely used medical imaging modalities and offers low-risk, real-time, and inexpensive bedside imaging^1^. An ultrasound image can be constructed from the time of flight and intensity of the received echoes produced by the scattering of acoustic waves from tissue inhomogeneities. Blood, however, is a poor ultrasound scatterer and the visibility of the blood pool can be enhanced through the intravenous injection of an ultrasound contrast agent (UCA)^2^. An UCA consists of a suspension of microbubbles that are highly echogenic owing to the large compressibility of their gas cores. The bubbles volumetrically oscillate in an ultrasound field and these oscillations produce a powerful nonlinear echo that contains harmonics of the driving frequency^3^. As such, bubble echoes can be discriminated from linear tissue echoes enabling the visualization and quantification of organ perfusion^4^.

To prevent bubble dissolution and coalescence, today’s UCA microbubbles are coated with a self-assembled mono-layer of phospholipids^5^. An additional merit of the shell is that it can be decorated with ligand molecules that avidly and specifically bind to target receptor molecules^6^. These targeted microbubbles can then be used for molecular ultrasound imaging to non-invasively diagnose and monitor endothelial-associated diseases such as inflammation and cancer^7–9^. Their biochemical targeting specificity can be combined to spatial (and temporal) specificity offered by the focusing of ultrasound to enable targeted drug delivery^10^. Indeed, volumetrically oscillating microbubbles can transiently permeabilize biological barriers allowing co-administered and bubble-loaded therapeutics to enter cells and tissues^11,12^. The combination of the excellent sensitivity of detecting a single bubble in vivo and the ability of a targeted microbubble to (i) bind to a specific biomarker, to (ii) locally deliver therapeutics, and to (iii) permeabilize biological barriers makes targeted microbubbles potential theranostic agents^13^.

To date, the available (pre-clinical) targeted microbubble suspensions have a polydisperse size distribution with diameters ranging from 1 to 10 µm^2,14^. The microbubbles are resonators with a characteristic resonance frequency that is inversely proportional to their size^15^. At resonance, the volumetric oscillation amplitude and the resulting nonlinear echo are at maximum. As such, only a small fraction of the bound bubbles resonates to the ultrasound field while even at a high dose of injected bubbles, only a small number of bubbles binds to the target site^16^. Thus, molecular ultrasound imaging would largely benefit from the increased sensitivity provided by a resonant bubble population driven at resonance^17–20^. Also in both radiation force assisted binding and radiation force enabled binding of targeted bubbles and targeted bubbles with a buried ligand architecture, respectively, a population of bubbles collectively tuned to resonance would be of interest as the radiation force is only acting on resonant bubbles^16,21^. Additionally, for quantitative molecular ultrasound imaging, it would be highly beneficial to acoustically discriminate bound bubbles from the freely floating and non-specifically bound ones, which can potentially be achieved through the spectral changes in the microbubble echo^22–24^. Finally, the efficacy and controllability of virtually all theranostic applications of targeted bubbles and ultrasound can be increased through the use of resonant monodisperse bubbles as they all rely on volumetric bubble oscillations that are governed by resonance^10^.

**Fig. 1:**
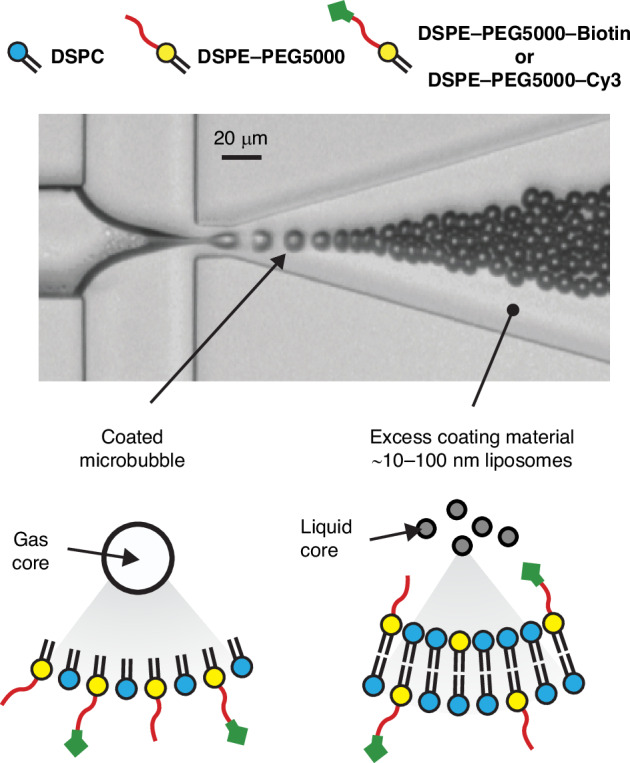
Monodisperse phospholipid-coated microbubbles were produced in a microfluidic flow-focusing device requiring a high excess coating material concentration. The produced bubbles were target-ready, i.e., ready for further functionalization though the inclusion of a Biotinylated PEG chain in the shell. To avoid bubble coalescence in the expanding nozzle of the flow-focusing device, high lipid concentrations are required resulting in a low coating efficiency where only 1 in every 10^4^ lipids adsorbs to a bubble interface. The remaining excess lipids are contained in 10 to 100-nm diameter liposomes that need to be removed from the bubble suspension to minimize the use of costly ligands during further bubble functionalization as well as to avoid competitive binding and blocking of the receptor molecules

Monodisperse microbubbles can be produced in a microfluidic flow-focusing device^25–27^. In such a device, a gas thread is focused between a co-flow through an narrow constriction where the gas thread destabilizes and pinches off releasing monodisperse bubbles at rates that can exceed 1 million bubbles per second (Fig. 1)^28^. The lipid coating material adsorbs to the gas-liquid interface already before pinch off^29,30^. The coating generally consists of a mixture of at least a primary phospholipid (e.g., DPPC, DSPC, DBPC) and a phospholipid attached to a long polymer polyethylene glycol (PEG) chain (e.g., DPPE-PEG5000 or DSPE-PEG5000)^5,31^. The PEGylated lipid provides stability against bubble coalescence via steric hindrance, and^5,30^, depending on the PEG concentration, it can provide stability against detection by the immune system and prevent undesired complement protein activation^32^. To produce targeted bubbles, targeting ligands can be coupled to the PEG chain^6,33^. This coupling can be performed before microbubble production^34^. However, stable coalescence free monodisperse microbubble production requires extremely high lipid concentrations such that only around 1 in every 10^4^ lipid molecules adsorbs to the bubble interface. This can be calculated by assuming a typical bubble concentration of 300 million bubbles per mL, a bubble radius of 3 µm, a total lipid concentration of 12.5 mg/mL, and an area per lipid molecule of 50 nm^2^ in the monolayer shell^35^. Thus, the majority of the coating material remains dispersed in the aqueous phase as lipid-vesicles that prevent coalescence through repulsive surface forces^30,36^. As targeting ligands are expensive, it is preferred to attach the ligands to the bubble surface after the bubbles have been produced and after the excess lipids are removed from the aqueous phase, a procedure often referred to as ’washing’. This approach thus requires a second washing step after bubble functionalization to remove any excess ligands in the liquid and thereby to avoid competitive binding and blocking of the receptor molecules.

Centrifugation is the standard method for washing the microbubble suspension^21,37–43^. The first step comprises dilution of the bubble suspension by 10 to 100 times, e.g., in a syringe or a 15 mL tube. Subsequently, the diluted bubble suspension is centrifuged at typically 100 to 300 g. After centrifugation, the supernatant containing the bubbles is pipetted into a separate container. The process of dilution and centrifugation usually has to be performed multiple times to sufficiently reduce the excess lipid concentration. As such, the method of centrifugation is laborious as well as user dependent due to its uncontrolled nature. Additionally, it is challenging to automate and integrate centrifugation into a monodisperse microbubble production facility. More importantly, during centrifugation, the microbubbles are subjected to a hydrostatic pressure increase of up to 100 kPa at 300 g, as we will show in Section III. Such overpressures typically reduce the surface area of the bubble by more than its threshold for collapse (buckling)^36,44,45^. Collapse of the monolayer shell is associated with lipid shedding, gas loss, and a reduced bubble stability^46^. Partial selective loss of the PEGylated lipid has also been suggested to occur^29^, which can lead to both a change in the acoustic and binding properties of the bubble. For all these reasons, it is of great interest to devise a fast, automated, controlled and user independent method for the removal of excess coating material while the bubbles are not subjected to an overpressure of more than several kPa.

In this work, we present a new method to remove excess coating material based on microfluidics and flotation that preserves the monodispersity of our target-ready monodisperse microbubbles. As the method relies on floatation, its use potentially extends beyond microbubbles, to microdroplets, particles, cells, and other entities with a different density than the medium in which they are suspended. Before we introduce the washing method in detail, we describe in Section II how the target-ready monodisperse microbubbles are produced by flow-focusing, and how we characterize the excess lipid concentration, the bubble size distribution, the functionality of the bubbles, and their acoustic response. In Section III, we investigate the traditional centrifugation method in detail. We calculate the overpressures that bubbles experience during centrifugation and by using our target-ready monodisperse microbubbles, we then demonstrate that centrifugation at typical accelerations leads to bubble instability and a decreased monodispersity. Then, in Section IV, we introduce our new washing method and discuss its design considerations as well as our fully automated implementation. We highlight the potential of our method by showing that the excess lipid concentration can be reduced in minutes by more than 4 orders of magnitude while the bubbles maintain the same monodispersity, functionality, and acoustic behavior as before applying the washing procedure. We end the paper with conclusions.

## Methods

### Monodisperse target-ready and fluorescently labeled microbubble production

Monodisperse microbubbles were formed in the flow-focusing device shown in Fig. 1a^36^. The temperature during bubble formation was 60^◦^C to minimize bubble coalescence in the outlet of the flow focusing device^47^. The lipid formulation comprised a 90:9:1 mixture of DSPC (Lipoid GmbH, Germany), DSPE-PEG5000 (BiochemPEG Scientific Inc., Watertown, USA), and either DSPE-PEG5000-biotin (BiochemPEG Scientific Inc., Watertown, USA) or DSPE-PEG5000-Cy3 (Broadpharm, San Diego, USA). The biotinylated DSPE-PEG5000 was employed to make the bubbles target-ready, i.e., ready for further functionalization after bubble production via a biotin–avidin coupling of for example an antibody. The DSPE-PEG5000-Cy3 (Cy3 is a fluorescent label) was used to characterize the reduction in excess coating material after microbubble washing, as will be described in Section II B. The total lipid concentration was 12.5 mg/mL in air saturated Isoton (Beckman Coulter). The microbubble filling gas consisted of a 85:15 volume-% mixture of CO_2_ and C_4_F_10_, respectively. A gas mixture was used to allow rapid and foam-free microbubble production as described in detail by Segers et al. ^48^. The produced microbubbles were collected in a capped 5 mL glass vial with a headspace pre-filled with C_4_F_10_ gas. During bubble collection, a vent needle ensured that the pressure inside the collection vial was equal to the atmospheric pressure, exactly as described by Segers et al. ^47^.

### Characterization of the excess lipid concentration

Characterization of the relative decrease in excess coating material after washing by either centrifugation or by our new washing method was performed by normalizing the fluorescence intensity of bubble-free medium after washing to that of the native bubble suspension. To this end, the fluorescent intensity was measured using a platereader (SpectraMax iD3, Molecular Devices). As the microbubbles disturb the platereader measurements, before each measurement, the bubbles were destroyed by putting the bubble sample in a sonicator bath for 3 min. Three 100-µL aliquots of each sonicated bubble sample were loaded in three wells of a 96-wellplate. The fluorescence intensity of each well was measured and averaged to reduce noise.

### Size distribution

The size distributions of the microbubble suspensions were measured using a Coulter Counter (Multisizer 4, Beckmann Coulter) with a 50 µm orifice. To this end, a 100 µL sample of a microbubble suspension was diluted in 100 mL Isoton (Beckmann Coulter).

### Fluorescence imaging

To validate that the Biotinylated PEGylated lipids stay in the microbubble shell during and after the washing procedure, a 0.7 mL sample of washed microbubbles was incubated for 5 mins with 1 µL of 15 µm streptavidin-oregongreen488 (Thermofischer). This resulted in approximately 40 streptavidin molecules for each biotin molecule. When the biotinylated lipids are present in the shell, the fluorescently labeled streptavidin will avidly bind to the microbubbles such that the microbubbles appear under fluorescence excitation. Before fluorescence imaging, the fluorescently labeled streptavidin was removed by washing the microbubble sample once more using the microfluidic washing method. Fluorescent microscope images were taken with a CCD camera (Grasshopper 3, FLIR) connected to a microscope with a LED light source (CoolLED, pE300 ultra).

### Acoustic attenuation spectra

For controlled targeted monodisperse bubble production, the acoustic response of the bubbles should not be affected by the washing procedure. Therefore, ultrasound attenuation spectra were measured of the native and the washed bubbles. To this end, a focused single element transducer was used to transmit 12-cycle ultrasound pulses with a 3 kPa peak negative acoustic pressure and a frequency between 0.5 and 5 MHz in steps of 50 kHz^18^. The first and last 3 cycles of each ultrasound pulse were Gaussian tapered. The transmit transducer (Olympus, V307) was pressure calibrated using a 0.2 mm needle hydrophone (Precision Acoustics). The choice of a pressure amplitude of only 3 kPa was motivated by the induced response of the microbubbles to be dominated by the viscoelastic properties of the bubble shell^3^. A second focused single element transducer (Olympus V304-SU) with its focal point co-aligned with the transmit transducer was used as the receive transducer. A sample holder containing a diluted microbubble suspension was placed in the mutual focal points of the two transducers. The attenuation coefficient *α* at transmit frequency *f*_*T*_ was calculated from the amplitude of the power spectrum at *f*_*T*_ obtained from a measurement with bubbles present and from that of a reference measurement without bubbles present, as follows:

1 $${\alpha }_{exp }=-\frac{10}{d}{\log }_{10}\frac{{\left|{V}_{{bub}}\left(\,{f}_{T}\right)\right|}^{2}}{{\left|{V}_{{ref}}\left(\,{f}_{T}\right)\right|}^{2}},$$

where |*V*_*bub*_(*f*_*T*_)|^2^ and |*V*_*re f*_ (*f*_*T*_)|^2^ are the amplitudes of the power spectra obtained from the time traces recorded with and without bubbles present at transmit frequency *f*_*T*_, respectively, and where *d* is the acoustic path length over which bubbles are present.

## Detailed analysis of the centrifugation method and its effect on mondisperse bubbles

The terminal rise velocity *v*_*t*_ of a microbubble with radius *R*_0_ relative to the surrounding liquid with viscosity *µ*_*l*_ and density *ρ* is obtained from a force balance of the buoyancy force and Stokes drag experienced by the rising bubble:

2 $${v}_{t}=\frac{2}{9}\frac{{\rho }_{l}g}{{\mu }_{l}}{R}_{0}^{2}.$$

**Fig. 2:**
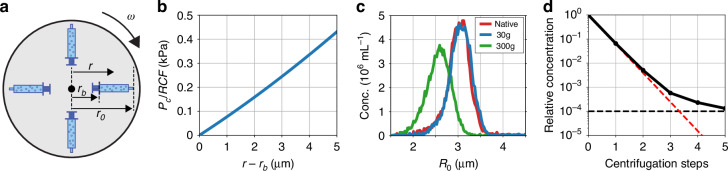
Microbubble washing by centrifugation is laborious and can induce bubble instability. **a** Microbubbles can be washed by centrifugation, i.e., through dilution and subsequent separation of the bubbles from the dilution medium^37^. **b** During centrifugation, the hydrostatic pressure in the bubble suspension increases proportionally with the relative centrifugal force (RCF), which is set on the bucket-rotor centrifuge (Beckman Coulter, Allegra X-12 with *r*_0_ = 207.8 mm and *r*_*b*_ = 157.8 mm, thus a total liquid column height of 5 cm). Using typical values of RCF between 100 and 300 for bubble washing results in a maximum hydrostatic overpressure during centrifugation between 30 and 100 kPa. **c** The hydrostatic pressure increase of 100 kPa during centrifugation at 300 g (RCF = 300) results in bubble instability and a 16% reduced bubble size. Only at a reduced RCF of 30 the bubble size distribution remains unaffected. D) Excess lipid concentration versus number of centrifugation steps. The plot shows that a relative concentration required for further functionalization (indicated by the black dotted line) and the use of targeted monodisperse microbubbles requires multiple centrifugation steps

For a microbubble with a 3 µm radius *v*_*t*_ is 20 µm/s in ambient gravity *g* = 9.81 m/s^2^. As such, it takes 1 h for this bubble to rise over a distance of 5 cm, which equals the height of our 10 mL syringe positioned vertically and loaded with 8 mL bubble suspension. In this calculation, the 0.5% decrease in hydrostatic pressure experienced by the rising bubble during its flotation over a distance of 5 cm and the corresponding 0.2% increase in bubble radius is deemed negligible. To accelerate floatation for microbubble washing, a centrifuge is typically used at a relative centrifugal force (RCF) of 100 to 300, i.e., at an acceleration of 100 to 300 times *g*^21,37–43^. The RCF during centrifugation is always expressed as the maximum RCF at the outer most radial position *r*_0_ in a bucket-rotor centrifuge, see Fig. 2a.

During centrifugation in a bucket-rotor centrifuge, the hydrostatic pressure *P*_*c*_ inside a column of liquid at depth *d* = *r* − *r*_*b*_ (see Fig. 2a) is given by:

3 $${P}_{c}\left(r\right)={\int}_{{\!r}_{b}}^{r}{\rho }_{l}{a}_{c}\left(r\right){dr},\,\,\text{with}\,\,{a}_{c}\left(r\right)={\omega}^{2}{r},$$

where *a*_*c*_ is the acceleration due to centrifugation and

4 $${\omega }^{2}=\frac{RCF\,g}{{r}_{0}}$$

is the angular frequency of the centrifuge. The solution of Eq. 4 is plotted in Fig. 2b, where *P*_*c*_ is normalized to the *RCF*. In the plot, an *r*_0_ of 207.8 mm and an *r*_*b*_ of 157.8 mm were used corresponding to the dimensions of our centrifuge (Beckman Coulter Allegra X-12). The figure shows that at the RCFs used for microbubble washing, i.e., within the range of 100 to 300 g, the microbubbles experience a maximum overpressure of 30 to 100 kPa.

For lipid-coated microbubbles, the decrease in bubble size as a function of overpressure can be calculated using the Marmottant model^44,45^. Calculations using typical shell properties, such as a shell elasticity of 1 N/m and an initial surface tension of 20 mN/m, show that the bubble size reduction depends on bubble size (see Supplementary Information). Specifically, a bubble with a radius of 0.5 µm decreases in size by 3%, while a bubble with a radius of 3 µm decreases in size by 16%. Additionally, even microbubbles with a radius of 0.5 µm buckle under an overpressure of 100 kPa. We hypothesize that shell buckling and the bubble surface area reduction during compression at 100 kPa, which amounts to nearly 30% for a bubble with a radius of 3 µm, lead to lipid loss and bubble dissolution. Therefore, we investigate the stability of our monodisperse 3-µm radius bubbles against the range of overpressures experienced during centrifugation. To this end, 500 µL of the native monodisperse microbubble suspension was diluted 16 times in 8 mL of air saturated Isoton (Beckman Coulter) and subsequently transferred to a 10 mL syringe. The syringe was then loaded in a centrifuge (Beckman Coulter, Allegra X-12). Two experiments were performed, the first one the typical RCF of 300 during 1 min of centrifugation and the second one at a lower RCF of 30 during 3 mins of centrifugation. The centrifugation time was thus adjusted to compensate for the rise velocity *v*_*r*_ of the bubbles, i.e., to ensure all bubbles reached the top of the syringe.

The results of the centrifugation experiment are shown in Fig. 2c demonstrating that the bubble size distribution becomes wider and the mean bubble size reduces when bubbles are centrifuged at 300 RCF. On the other hand, when the RCF is reduced to 30, which is below typical accelerations employed for bubble washing, the size distribution remains unaffected. Indeed, buckling of monodisperse microbubbles with a radius of 3 µm is expected at an overpressure that exceeds 20 kPa^36^. The centrifugation results suggest that when the maximum hydrostatic pressure during centrifugation is kept below the buckling pressure, the bubble size distribution remains unaffected.

We now turn our attention to the excess lipid concentration. Figure 2d shows that indeed, after a single centrifugation step, the relative concentration of lipids in the bubble sample reduced by the dilution factor of 16 (indicated by the red dotted line in 2D). Similarly, after a second centrifugation step performed on the same bubble sample, the excess lipid concentration decreased by the expected factor of 16^2^, and so on. After 4 to 5 washing steps on the same bubble sample, the excess lipid concentration plateaus, which can be explained by the loss of shell material from the unstable bubbles during centrifugation. Thus, centrifugation can negatively affect the bubble size distribution and repeated centrifugation steps are required to sufficiently reduce the excess lipid concentration, which are prone to user variability.

## Microfluidic method for removal of excess coating material

### Working principle: rapid floatation at the small scale

Physical forces that may be employed for excess coating removal, i.e., to separate microbubbles from the bulk liquid include the buoyancy force, hydrodynamic forces in microfluidic channels (e.g., inertial forces), or optical and acoustical radiation forces^49^. Steric hindrance can be employed as well, for example in a cross flow filtration device with a micropore filter^50^. The deterministic lateral displacement method to passively force microbubbles into a clean stream of liquid using a microfluidic grid of pillars may be exploited as well for bubble washing^51^. However, for the intended application of removing excess coating material in a microbubble production facility while keeping a low overpressure, the *most* simple and robust method is preferred. Flotation is simple and in principle passive. Whereas floatation at the centimeter scale needs to be accelerated by centrifugation, here, we demonstrate that flotation at the millimeter scale can be integrated in a microfluidic device for fast and fully automated removal of excess coating material within minutes.

### Microfluidic implementation

**Fig. 3:**
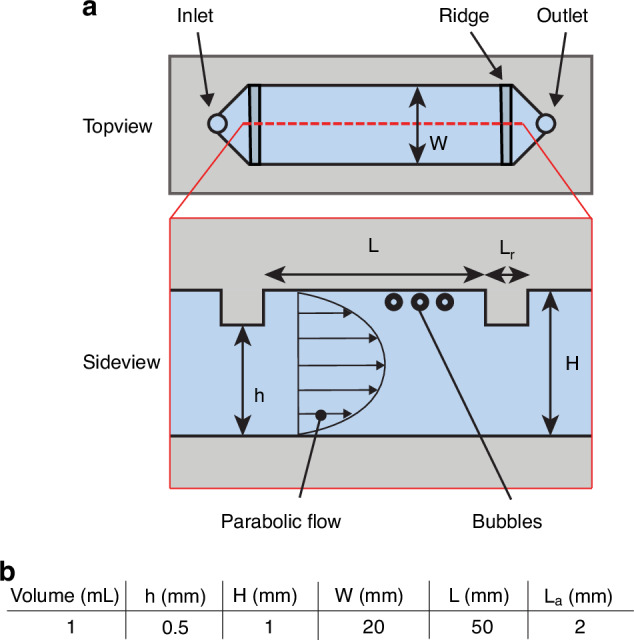
Schematic of the microfluidic washing chip. **a** Top and side view of the microfluidic washing chip. Microbubbles float against the top of the channel while a different medium free of coating material is pumped through the channel. The bubbles remain trapped between the ridges. **b** Table containing the dimensions of the microfluidic chip

The microfluidic washing chip (Fig. 3) includes a channel with a height H of 1 mm, specifically designed to wash 3-µm radius bubbles (v_t_ of 20 µm/s) as they float across the channel height within a minute. To redesign H for bubbles of a different size, Eq. 2 can be multiplied by the maximum time allowed for floatation:

5 $$H={v}_{t}{t}_{\max}.$$

For instance, to wash 0.5-µm radius bubbles (v_t_ of 0.5 µm/s) within 5 min, an H of ≤ 150 µm is required, which remains easy to fabricate. The smallest R_0_ that can be washed using the present method can be estimated from the Peclet number based on buoyancy relative to thermal diffusion:

6 $${Pe}=\frac{{v}_{t}H}{D}.$$

Here, *D* = *k*_*B*_*T* (6*πµ*_*l*_*R*_0_)^−1^ is the thermal diffusivity of the bubble according to the Stokes-Einstein-Sutherland equation, with kB Boltzmann’s constant and T the absolute temperature. Using Eq. 5, 6, and a t_max_ of 5 min, it is straightforward to calculate that only for bubble radii below 0.2 µm the Peclet number drops below unity, i.e., for R_0_ < 0.2 µm thermal diffusion significantly affects microbubble floatation.

The total internal volume of the channel (*W* × *L* × *H*) is easily scalable and sets the total volume of bubble sample that can be washed at once. In the present implementation the total volume was set to 1 mL, containing approximately 300 million bubbles as derived from our typical bubble concentration obtained with the flow focusing device detailed in Section II A (300 million bubble/mL). Once the bubbles reach the top wall of the channel through passive flotation, a constant flow of clean liquid is pumped through the channel thereby transporting the excess coating material downstream. To maintain the bubbles inside the chip, a physical barrier is introduced in the top wall of the channel, indicated by ridge in Fig. 3a. The formation of a recirculation zone behind the sharp edge of the ridge can occur at Reynolds numbers above unity. Such a recirculation zone may counteract floatation and in a more extreme case, result in the detachment of bubbles from the channel ceiling. For the channel dimensions shown in Fig. 3b, the Reynolds number is larger than unity at flowrates above 0.5 mL/min. As the length of such a recirculation zone is of the same order of magnitude as the size of the restriction^52^, the effect of recirculation is minimized by limiting the height of the physical barrier with respect to the channel length, i.e., H − h ≪ L. Additionally, to limit the drag force on the bubbles, which may induce their downstream translation, the shear rate at the bubble location is limited by ensuring fully developed flow instead of plug flow inside the channel. To this end, the channel inlet and outlet are tapered at a 30^◦^ angle to aid in developing the flow. Additionally, the channel length *L* was set at a length of 4 times the channel width *W*. An aspect ratio of 4 was selected because it allows the flow to fully develop while the Reynolds number and the pressure drop across the channel remain low. However, we stress that the exact width to length ratio, as well as the channel height are all not critical, i.e., the washing method is robust across a large variation in channel dimensions.

The robustness of the washing method is also reflected in the simple channel geometry with feature dimensions of the order of 1 mm that can be easily fabricated using for example micromilling, 3D printing, hot embossing, and double sided tape. In this work, the microfluidic washing chip was fabricated using a milling machine with a 1 mm diameter mill in a 9-mm thick block of PMMA (polymethyl methacrylate). To close the channels, a PET (polyethylene terephthalate) film was attached to the PMMA block using 70-µm thick double sided tape from which the channel shape was cut out before it was bonded on the PMMA block.

The dimensions of the washing chip presented in this work are given in Fig. 3b. Using the Hagen-Poiseuille equation, and by summing over the hydraulic resistances of each channel section, it can be estimated that at a pressure drop of 1 kPa, the flow rate through the washing chip is approx. 1.2 L/min. As such, the entire volume of the washing chamber can be replaced within milliseconds. Due to this short timescale of replenishment, two regimes of material efflux are expected. On the short convection dominated timescale (ms-s), the excess coating material present in the bulk is rapidly flushed out. On the longer timescale of the diffusion of the 30 nm lipid vesicles, excess material in the boundary layer between and around the microbubbles is expected to diffuse along the concentration gradient directed toward the depleted center region of the channel. The timescale for diffusion of lipid vesicles out of the fluid layer one bubble diameter in thickness can be estimated from one dimensional Fickian diffusion (*t* = *l*^2^(4*D*)^−1^ where *l* ≈ 10 µm) to be 4 s. However, here, a constant material source is assumed, which obviously does not hold when the excess material concentration reduces over time. As such, the diffusion time scale for loss of excess coating material out of the stagnant fluid layer between the bubbles located at the top wall of the washing chamber will most likely be much longer. Thus, for excess coating material removal using the present washing chip diffusion is expected to play a role, which we will further investigate in the Section IV D.

### Automated procedure to remove excess coating material with the washing chip

**Fig. 4:**
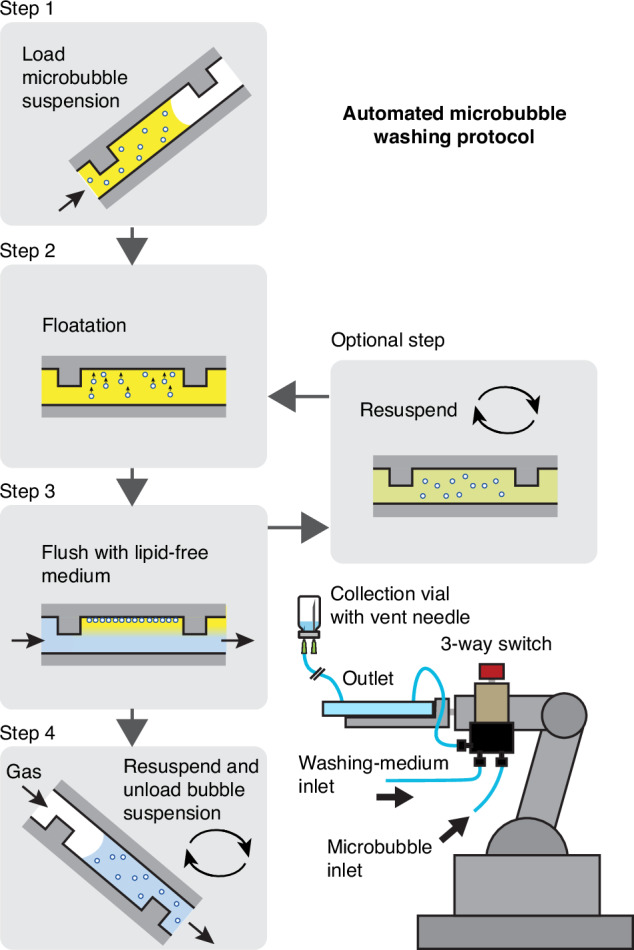
Developed protocol to remove excess lipids from a sample of microbubbles. First, the microbubbles are loaded while the washing chip is positioned under a 30° angle to remove all the gas. Then, the microbubbles are allowed to float to the top wall. In step 3, clean coating material-free medium is pumped through the chip to take away the excess coating material. Optionally, the bubbles can be resuspended to increase the diffusion-limited removal of excess coating material in the boundary layer between the bubbles. Finally, the washed microbubble suspension is unloaded from the chip using pressurized gas while it is held under a 45° angle. The entire procedure was fully automated by mounting the microfluidic chip on a 3D-printed robotic arm containing 3 servo motors

The washing procedure detailed in what follows can be entirely performed by hand. However, we now demonstrate that it can also be fully automated to enable controlled (bedside) production of microbubbles. To this end, the microfluidic washing chip was mounted on a 3D-printed arm containing 3 servo motors, see bottom right in Fig. 4. The servo motors can control the position of the chip and its movement, e.g., to rotate the chip in order to re-disperse the floating microbubbles. The inlet of the washing chip was connected to a fluidic 3-way switch (CIL-V-101L, IDEX) with a low internal volume. The switch is used to either connect the inlet of the washing chip to a syringe with the native bubble suspension, or to a syringe with the clean medium that is used to wash the microbubbles. The protocol to operate the washing chip was programmed in an Arduino controller and it contains the following steps, also visually shown in Fig. 4:

#### Step 1: loading

The microbubble suspension is loaded from the bottom inlet into the microfluidic washing chamber that is positioned under a ∼30^◦^ angle with respect to gravity to ensure all the air is pushed out of the washing chip during microbubble loading.

#### Step 2: waiting

A waiting step of 1 min allows the bubbles to float to the top of the channel.

#### Step 3: washing

Clean coating material-free medium is pumped through the channel to take away any excess coating material while the bubbles remain positioned in the chip against the top wall. The flow rate and the total volume pumped through the washing chip are the control parameters that will be investigated in the next section.

#### Optional step

Optionally, a resuspension step can be incorporated with the intention to homogeneously disperse the coating material present in the fluid layer close to the channel wall around the bubbles throughout the medium. After resuspension through rotation at 1 Hz of the chip around its longitudinal axis, step two is again followed to allow the microbubbles to float to the top wall before in additional step 3, the excess coating material is further removed.

#### Step 4: unloading

To unload the microbubbles, they are first resuspended by rotating the chip around its longitudinal axis at a frequency of 1 Hz. Subsequently, the washing chip is held under a 45^◦^ angle and a slug of gas is slowly infused in the washing chip from the bottom while the microbubble suspension leaves the chip from the top outlet into a vial. The gas can be the same as the microbubble filling gas to ensure no gas exchange during bubble washing^53^.

### Characterization of the automated microbubble washing procedure

To characterize the washing procedure, 1 mL of a monodisperse fluorescently labeled microbubble suspension with a mean bubble radius of 3.1 µm (see microbubble size distribution in Fig. 6) was loaded in the washing chip. Then, the bubbles were left to float to the top wall of the device for 1 min. Subsequently, air saturated coating material-free Isoton was pumped through the washing chamber while 0.5-mL aliquots were collected at the outlet in 1-mL glass vials at selected time intervals. The relative concentration of excess coating material in each aliquot was measured as before. To elaborate on the role of diffusion in excess coating material removal, the experiment was performed at a flow rate of 0.5 and 5.0 mL/min.

**Fig. 5:**
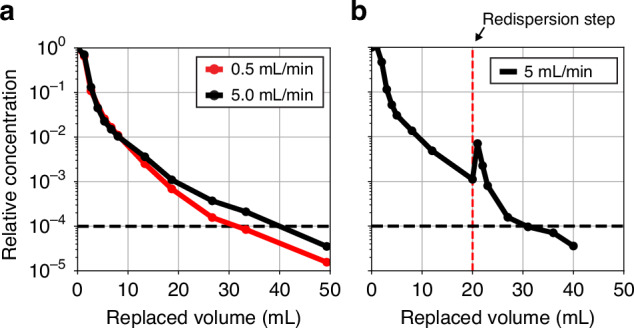
The concentration of excess coating material reduces by ten thousand times when 30 to 40 mL of coating material-free medium is pumped through the washing chip. **a** Relative concentration of excess lipids present in the efflux liquid of the washing chip plotted as a function of the liquid volume pumped through the washing chip. The experiment was performed at a flow rate of 0.5 mL/min (red curve) and 5.0 mL/min (black curve). **b** Relative excess lipid concentration as a function of replaced volume where a redispersion step was performed halfway the washing procedure

The measured excess lipid concentration relative to the concentration in the native bubble suspension is plotted in Fig. 5a as a function of the liquid volume pumped through the washing chamber. Note that the slope of the curves decreases with an increase in replaced volume. Initially, the efflux of excess coating material is dominated by advection. At a later stage, when the excess coating material from the major part of the washing chamber has been depleted, diffusion of the lipid vesicles in the fluid layer close to the channel walls starts to play a role. This is also evident from the two curves obtained at the different flow rates that start to deviate when the replaced volume exceeds 10 mL. The observed difference is due to the 10 times longer duration of the washing experiment for the lower 0.5 mL/min flow rate allowing, at the same amount of replaced volume, more lipid vesicles in the fluid layer around the bubbles to diffuse toward the center of the washing chamber thereby promoting their advective efflux. Nevertheless, both curves show that the excess lipid concentration can be reduced by more than 10^4^ times when 34 mL and 40 mL of clean liquid is pumped through the washing chamber at 0.5 and 5.0 mL/min, respectively, corresponding to a washing time of 68 and 8 min, respectively.

To reduce the total time required for the washing procedure, we implemented a re-dispersion step (Fig. 4). Once 20 mL of clean Isoton was pumped through the washing chip at a flow rate of 5 mL/min, the liquid flow was stopped, and the chip was rotated back and forth at 1 Hz around its longitudinal axis for 30 s to re-disperse both the bubbles and the lipid vesicles. After a waiting time of 1 min allowing the bubbles to float against the top wall, the liquid flow was resumed at a flow rate of 5 mL/min. Note the sudden increase in the coating material concentration in the efflux liquid showing that indeed, diffusion of the lipid vesicles from the channel walls toward the channel center is the limiting factor in the time required for the washing procedure.

### Characterization of the washed microbubbles

**Fig. 6:**
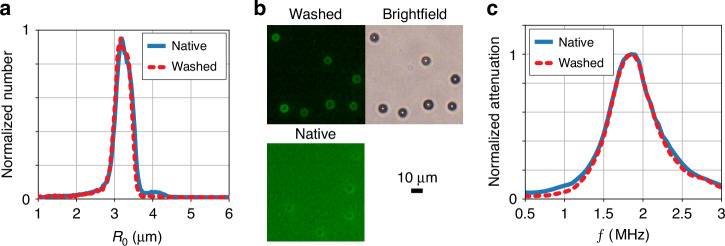
Our microfluidic washing method does not affect the size distribution, functionality, and acoustic response of washed monodisperse microbubbles. **a** Microbubble size distribution measured before and after washing. **b** Fluorescent image of fluorescently labeled streptavidin attached to microbubbles after they had been washed. The top right image shows the corresponding brightfield image. The background signal in the washed image is much reduced with respect to that in the native bubble suspension (bottom left). **c** Ultrasound attenuation spectra of the microbubbles before and after washing using the microfluidic chip

Figure 6a shows the size distribution of monodisperse 3.1-µm radius target-ready microbubbles before and after they were washed. The washing procedure was performed exactly as in Fig. 5a at a flow-rate of 5.0 mL/min. The figure demonstrates that the washing chip and its operating procedure devised in the present work do not affect the size distribution of microbubbles. The size distributions were normalized to highlight their similarity. The bubble concentrations did not vary more than 10%, which is within the experimental uncertainty involved in the pipetting and withdrawal of bubbles from the collection vial.

To verify that PEGylated lipids remained in the bubble shell over the washing procedure, fluorescent streptavidin was added to washed biotinylated microbubble sample. The microbubbles were washed as before, at a flow rate of 5.0 mL/min and a total replaced volume of 40 mL. After streptavidin incubation, the microbubbles were washed once more to remove excess streptavidin. Figure 6b shows a fluorescence image (top left) along with a brightfield image (top right) of the same field of view. The microscopy images show that each bubble was biotinylated, and, based on the low background fluorescence signal compared to an unwashed sample (bottom right), that the excess coating material was successfully cleared. The same procedure was repeated with bubbles that were not biotinylated, and no fluorescence was detected (bottom left).

Finally, the acoustic resonance behavior of the native and the washed target-ready microbubbles were characterized using narrowband acoustic attenuation measurements. The attenuation curves shown in Fig. 6c are nearly indistinguishable showing that both the resonance frequency as well as the damping (as derived from the width of the attenuation curves) were not affected by the washing procedure. As such, since the size did not change, the washing procedure did not induce any change in viscoelastic properties of the lipid shell.

## Conclusions and outlook

A novel method for fast, controlled, and fully automated removal of excess coating material from a microbubble suspension was presented. The method is based on flotation at the millimeter scale in a microfluidic washing chamber. We demonstrate that the washing procedure can lower the excess lipid concentration by 4 orders of magnitude while the bubble size distribution, functionality, and acoustic response remain unaffected.

The presented method is a potential gateway to the fully automated production of functionalized monodisperse microbubbles for improved molecular ultrasound imaging and therapy. Where industrial scale production of monodisperse bubbles using microfluidics remains a challenge due to cross talk in parallelized devices^54^, a single flow-focusing device can produce a clinical dose of 300 million bubbles in 5 min, which potentially allows for the on-demand production of microbubbles at the bedside. Our microbubble washing method may be easily incorporated in a bedside bubble production facility. GMP (good manufacturing practice) compliance may be achieved by incorporating a flow-focusing device, all liquids, and the washing chamber in a sterile cartridge.

As a future perspective, the acoustic response of single functionalized bubbles needs to be characterized both in the free field and upon their molecular binding to a boundary to investigate (i) whether their acoustic response is uniform in the free field, (ii) how their response changes upon binding, and (iii) whether the change in acoustic response is uniform across a population of monodisperse bubbles. Recently, we have shown that a uniform acoustic response of microfluidically formed monodisperse microbubbles is not self-evident^55^. Monodisperse microbubbles can have non-uniform viscoelastic shell properties and thereby a non-uniform acoustic response. The microbubble washing method presented in this work now allows to study the role of shell formulation and microfluidic production parameters on the acoustic uniformity of functionalized microbubbles.

### Supplementary information

Supplementary information
