# Supplementary material for: Functionalized monodisperse microbubble production: microfluidic method for fast, controlled, and automated removal of excess coating material
Source: Microsyst Nanoeng. 2024 Aug 30;10:120. doi: 10.1038/s41378-024-00760-y (PMC11364838; doi:10.1038/s41378-024-00760-y)
Supplement: Supplementary file 1 — Supplementary information [file 41378_2024_760_MOESM1_ESM.pdf]

Supplementary Information for 'Functionalized monodisperse microbubble production: Microfluidic method for fast, controlled, and automated removal of excess coating material'

M.R.P. van den Broek,[1] M. Versluis,[2] A. van den Berg,[1] and T. Segers[1]

[1]*BIOS / Lab on a Chip Group, Max Planck Center Twente for Complex Fluid Dynamics, University of Twente, Enschede, The Netherlands*

[2]*Physics of Fluids Group, University of Twente, Enschede, The Netherlands*

(*Electronic mail: t.j.segers@utwente)

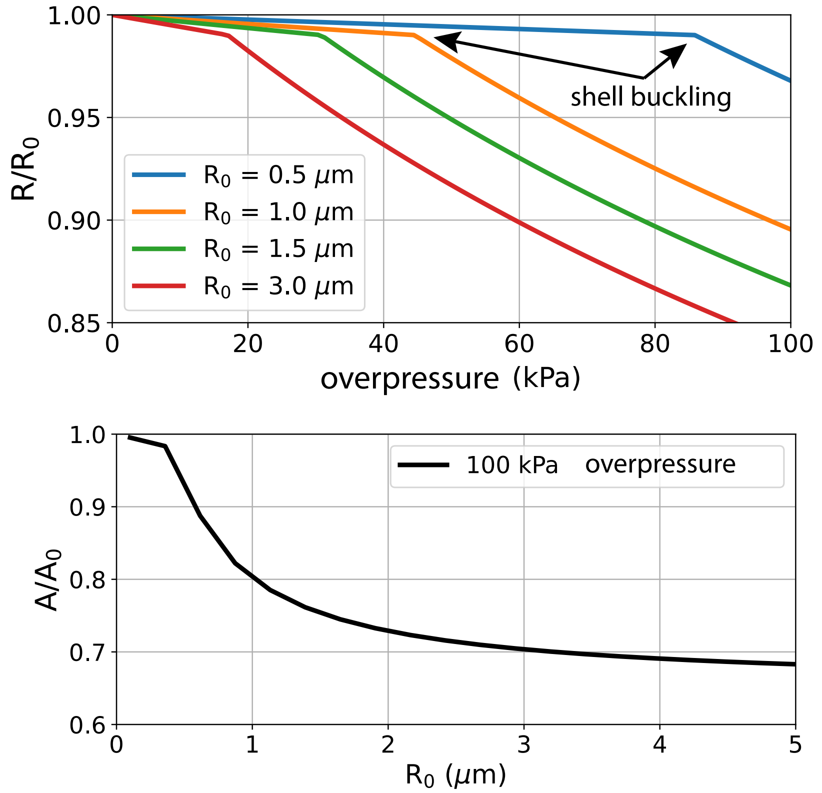

FIG. S1. (top) Bubble radius R normalized by the bubble radius at zero overpressure R_0 as a function of overpressure for 4 different bubble radii. (bottom) Normalized bubble surface area decrease at an overpressure of 100 kPa as a function of bubble size. The shell parameters in the modeling were a shell elasticity of 1 N/m and an initial surface tension of 20 mN/m.
